# Pulmonary embolism in patients with coronavirus disease-2019 (COVID-19) pneumonia: a narrative review

**DOI:** 10.1186/s13613-020-00741-0

**Published:** 2020-09-16

**Authors:** Yasser Sakr, Manuela Giovini, Marc Leone, Giacinto Pizzilli, Andreas Kortgen, Michael Bauer, Tommaso Tonetti, Gary Duclos, Laurent Zieleskiewicz, Samuel Buschbeck, V. Marco Ranieri, Elio Antonucci

**Affiliations:** 1 Dept. of Anesthesiology and Intensive Care Medicine, Jena University Hospital, Am Klinikum 1, 07743 Jena, Germany; 2 Intermediate Care Unit, Emergency Department, Ospedale Guglielmo da Saliceto, Piacenza, Italy; 3 Service d’Anesthésie et de Réanimation, Aix Marseille Université, Assistance Publique Hôpitaux Universitaires de Marseille, Hôpital Nord, Marseille, France; 4 Dipartimento di Scienze Mediche e Chirurgiche, Anesthesia and Intensive Care Medicine, Alma Mater Studiorum, Università di Bologna, Policlinico di Sant’Orsola, Bologna, Italy

**Keywords:** SARS-CoV-2, COVID-19, Pulmonary embolism, Thromboprophylaxis, Venous thromboembolism

## Abstract

**Background:**

Preliminary reports have described significant procoagulant events in patients with coronavirus disease-2019 (COVID-19), including life-threatening pulmonary embolism (PE).

**Main text:**

We review the current data on the epidemiology, the possible underlying pathophysiologic mechanisms, and the therapeutic implications of PE in relation to COVID-19. The incidence of PE is reported to be around 2.6–8.9% of COVID-19 in hospitalized patients and up to one-third of those requiring intensive care unit (ICU) admission, despite standard prophylactic anticoagulation. This may be explained by direct and indirect pathologic consequences of COVID-19, complement activation, cytokine release, endothelial dysfunction, and interactions between different types of blood cells.

**Conclusion:**

Thromboprophylaxis should be started in all patients with suspected or confirmed COVID-19 admitted to the hospital. The use of an intermediate therapeutic dose of low molecular weight (LMWH) or unfractionated heparin can be considered on an individual basis in patients with multiple risk factors for venous thromboembolism, including critically ill patients admitted to the ICU. Decisions about extending prophylaxis with LMWH after hospital discharge should be made after balancing the reduced risk of venous thromboembolism (VTE) with the risk of increased bleeding events and should be continued for 7–14 days after hospital discharge or in the pre-hospital phase in case of pre-existing or persisting VTE risk factors. Therapeutic anticoagulation is the cornerstone in the management of patients with PE. Selection of an appropriate agent and correct dosing requires consideration of underlying comorbidities.

## Introduction

Since the emergence of coronavirus disease-2019 (COVID-19) as a result of infection with Severe Acute Respiratory Syndrome Coronavirus 2 (SARS-CoV-2) [1], several reports have described significant procoagulant events, including life-threatening pulmonary embolism (PE), in these patients [2–45]. Abnormalities of various coagulation parameters were frequently reported [46, 47] and have been linked to poor prognosis [48]. Unfortunately, little is known about the epidemiology and the pathophysiologic mechanisms underlying COVID-19-associated PE because of the lack of large prospective studies in this context. Understanding these aspects is crucial for the early diagnosis and appropriate management of this potentially fatal complication. In particular, the optimal dosage and duration of prophylactic anticoagulation are major concerns. Indeed, PE was reported to occur in critically ill COVID-19 patients despite thromboprophylaxis [31], questioning the possible role of the implementation of higher dosage of thromboprophylaxis than those used in the standard practice. In this report, we review the current literature on the subject to define the epidemiology, possible underlying pathophysiologic mechanisms, and therapeutic implications of PE in relation to COVID-19.

## Epidemiology and outcome of PE in COVID-19

As of May 24, 2020, 27 case reports describing the clinical characteristics of 30 patients with COVID-19-associated PE have been published (Table 1) [2–28]. The median age of these patients was 59 years (interquartile range (IQ) 44–68 years, range 17–84 years) and 18/30 patients were male. One-third of the cases had no comorbid conditions prior to ICU admission. There was no detectable source of PE in most of the cases (24 patients, 80%). Diagnosis of PE was made at a median of 11 days (IQ: 7–17, range: 4–22 days) from the onset of SARS-CoV-2 symptoms. In 20 patients (66.7%), PE was bilateral; the majority of cases were treated with LMWH. Only 8 patients (26.7%) had central PE, of which 2 patients died.

**Table 1:** Published case reports describing patients with COVID-19 complicated by pulmonary embolism (PE)

Authors (country)	Sex, age (years)	Time to PE (days)*	Comorbid conditions	Source of PE	Extent of PE	Therapy	Outcome, remarks
Danzi et al. (Italy) [2]	F, 75	10	None	None	Bilateral	LMWH	NR
Cellina et al. (Italy) [3]	M, 60	13	Overweight	None	Bilateral; left main pulmonary artery and right interlobar artery	NR	NR
Ullah et al. (USA) [4]	F, 59	> 8	Hypertension, type 2 diabetes mellitus	None	Bilateral; central and proximal segmental pulmonary artery and linear sellar PE	LMWH → Apixaban	Discharged after 1 week
Casey et al. (USA) [5]	M, 42	12	None	None	Bilateral segmental; infarct in the right lower lobe	LMWH	Discharged home
Foch et al. (France) [6]	M, 50	7	Recent long-haul flight	None	Middle lobe and segmental	LMWH	NR
Rotzinger et al. (Switzerland) [7]	M, 75	4	None	None	Right middle lobar segmental	LMWH	NR
Fabre et al. (France) [8]	F, 45	7	Obesity, hypertension	Clot in patent foramen ovale, DVT of left leg	Massive bilateral proximal PE	Surgical embolectomy, ECMO	Death
Sulemane et al. (UK) [9]	M, 60	–	Hypertension, hypercholesterolemia	Small, highly mobile mural thrombus within RV free wall	Bilateral; inferior lingula and segmental branches to lateral segment of middle lobe	Thrombolysis	NR
Audo et al. (Italy) [10]	M, 59	> 10 days	None	None	Massive bilateral; right atrium and left and right main pulmonary arteries	Surgical embolectomy	Transferred to a regular ward
Le Berre et al. (France) [11]	M, 71	17	None	Thrombosis of right posterior tibial vein	Anterior basal branch of right inferior lobe pulmonary artery	LMWH	Survived
Jafari et al. (Iran) [12]	F, 50	7	None	None	Large saddle PE	Heparin and antithrombotic treatment	Discharged home
Griffin et al. (USA) [13]	M, 52	18	Smoker	None	Bilateral	LMWH → rivaroxaban	Discharged receiving supplemental oxygen
	F, 60	18	Ovarian cancer post oophorectomy, DVT 18 years earlier	None	Multiple bilateral segmental and subsegmental PE	LMWH → rivaroxaban	Discharged receiving supplemental oxygen
	M, 68	22	Hypertension, diabetes mellitus	None	Bilateral	LMWH	Favorable outcome
Martinelli et al. (Italy) [14]	F, 17	9	Obesity, pregnancy	None	Segmental PEin the right superior lobe	LMWH	Discharged home Urgent cesarean sections (29W)
Lushina et al. (USA) [15]	M, 84	14	Hypertension	None	Bilateral lobar PE	LMWH; thrombectomy	Death on day 2
Harsch et al. (Germany) [16]	F, 66	> 7	Atrial fibrillation	None	Bilateral pulmonary arterial emboli in the lower lobes	Apixaban	Discharged home
Ueki et al. (Switzerland) [17]	M, 82	7	None	None	Thrombus in right pulmonary artery	NR	NR
Ioan et al. (Spain) [18]	M, 61	7	Smoking, hypertension	None	Bilateral	r-tPA	NR
Bruggemann et al. (Netherland) [19]	M, 57	14	Peripheral arterial disease	None	Multiple PE in the right pulmonary artery and bilateral (sub)segmental PE	LMWH	NR
Perez-Girbes (Spain) [20]	M, 68	NR	NR	NR	Right lobar PE and segmental PE in the right superior lobe	NR	NR
Khodamoradi et al. (Iran) [21]	F, 36	5	Pregnancy, 5 days after cesarean section	None	Right side interlobar artery, posterior basal segment, and the lingular branch	LMWH	Discharged home
Poggiali et al. (Italy) [22]	M, 64	27	None	DVT	Left subsegmental PE	Fondaparinux/dapigatran	Discharged home
Marsico et al. (Spain) [23]	M, 32	14	None	None	Bilateral segmental and subsegmental branches of pulmonary arteries	LMWH	Discharged home
	F, 59	19	Hypertension, hypothyroidism	None	Bilateral segmental and subsegmental branches of pulmonary arteries.	LMWH	Discharged home
Schmiady et al. (Swizerland) [24]	F, 54	3	HIT-II	Multiple thrombi in the inferior vena cava, the right atrium, and the pelvic veins	Central pulmonary artery with occlusion of the lower right and middle pulmonary artery	Argatroban Thrombectomy ECMO	NR
Polat and Bostancı (Turkey) [25]	F, 41	NR	Diabetes mellitus	None	Bilateral central PE	r-tPA/heparin	Sudden death
Ahmed et al. (UK) [26]	F, 29	14	Diabetes mellitus, obesity, pregnancy	None	Right lower lobe	NR	Death
Molina et al. (USA) [27]	M, 23	NR	Nitrous oxide abuse	DVT	Saddle PE	r-tPA	NR
Vitali et al. (Italy) [28]	M.70	22	None	None	Bilateral lobar and segmental	LMWH	Discharged home

DVT: deep venous thrombosis, F: Female, LMWH: low-molecular weight heparin, HIT: heparin-induced thrombocytopenia, M: male, NR: not reported, PE: pulmonary embolism, r-tPA: recombinant tissue plasminogen activator, UK: United Kingdom

* Since the initial SARS-CoV-2 symptoms

Few cohort studies have reported the epidemiology of PE in COVID-19 patients, irrespective of the severity of illness and need for hospital admission (Table 2). Most of these were retrospective cohorts [29, 30, 32–37, 39–42, 44] and probably underestimated the incidence of PE because of the lack of a systematic approach to screening for this complication. The epidemiologic estimates were also likely influenced by the relatively short follow-up periods and different severities of infection. In two large retrospective French cohorts, the incidence of PE among patients positive for SARS-CoV-2, regardless of whether they were or were not admitted to hospital, was 1.1 and 3.4%, respectively [29, 30]. Evidence of PE was found in 23–30% of patients who underwent CTA imaging. Interestingly, comorbid conditions were similar in patients with PE and those without [29, 30]. We may speculate, therefore, that the occurrence of PE maybe related, at least in part, to the progression and severity of COVID-19 illness and not only to the physiologic status prior to infection. Indeed, patients with COVID-19 infection and PE had higher d-dimer levels, indicating higher severity of illness and more pronounced inflammatory response, than those without PE [30].

**Table 2:** Summary of cohort studies reporting the epidemiology and outcome of thromboembolic complications in patients with COVID-19

Authors, year Country	Design	Number of patients	Incidence of PE	Remarks
Grillet et al. France [29]	Retrospective study SARS-CoV-2 according to + ve RT-PCR or high clinical suspicion	SARS-CoV-2 + ve: 2003 pts Hosp. adm..: 280 pts CTA performed: 100 pts	23% (among patients with CTA) 8.9% (among hosp. admissions) 1.1% (among all COVID-19 + ve pts)	Radiologic study, no clinical correlates Average time to CTA: 12 days PE pts.: ICU admissions, 74%, MV: 65% No differences in comorbidities between PE and no PE Selection bias (only severe cases/clinical deterioration with CTA)
Leonard-Lorant et al. France [30]	Retrospective study 2 French hospitals	SARS-CoV-2 + ve: 961 pts COVID-19 with CTA: 106 pts (97 + ve RT-PCR, 9 high clinical suspicion)	30% (among patients with CTA) 3.4% (among SARS-CoV-2 + ve pts)	PE pts.: ICU admissions, 75% PE: 22% main PA, 34% lobar, 28% segmental, 16% subsegmental No differences in comorbidities between PE and no PE Selection bias (only severe cases/clinical deterioration with CTA) d-Dimer levels associated with PE
Helms et al. France [31]	Prospective cohort 4 ICUs in 2 hospitals	150 pts	16.7%	Short follow-up in some patients (7 days) PE mostly men (24/25, mean age 62 years old) PE: 36% main PA, 32% lobar, 20% segmental and 12% subsegmental PE: detected at a median of 5.5 days after ICU admission Thromboembolic events more common in COVID-19 ARDS compared to historic ARDS cohort All patients received at least standard dose thromboprophylaxis
Klok et al. Netherlands* [32]	Retrospective cohort ICUs in 3 hospitals	184 pts	13.6%	31% thrombotic complications Age and coagulopathy were independent predictors of thrombotic complications Median duration of follow-up per patient was 7 days All patients received at least standard doses thromboprophylaxis
Lodigiani et al. Italy [33]	Retrospective single-center cohort	388 pts (61 ICU pts)	2.6% overall 4.2% (of 48 closed ICU cases)	Thromboprophylaxis was used in 100% of ICU patients and 75% of those on the general ward Incidence may have been highly under-estimated due to the low number of specific imaging tests performed
Llitjos et al. France [34]	Retrospective cohort 2 ICUs	26 pts	23%	Duplex ultrasound performed as standard of care 31% (*n* = 8) of prophylactic anticoagulation and 69% (*n* = 18) of therapeutic anticoagulation
Poissy et al. France [35]	Retrospective cohort ICU	107 pts	20.6%	PE occurred within a median 6 days after ICU admission Despite a similar severity on admission to the ICU, the frequency of PE in COVID-19 patients was twice higher than the frequency in the control period and in 40 influenza patients All patients received at least standard doses thromboprophylaxis Low number of associated DVTs d-Dimer levels, plasma factor VIII activity, and factor Willebrand antigen levels were associated with a greater PE risk
Beun et al. Netherlands [36]	Retrospective cohort ICU	75 pts	26.6%	High-dose UFH of more than 35,000 IU/day reported in 4 patients with PE due to heparin resistance Factor VIII, fibrinogen, and d-dimer levels were elevated, while almost all of the antithrombin levels were in the normal range in all patients
Middeldorp et al. Netherlands [37]	Retrospective single-center cohort COVID-19 according to +ve RT-PCR or high clinical suspicion	198 pts (75 ICU)	6.6% overall 15% ICU	All patients received at least standard doses thromboprophylaxis Median follow-up duration was 15 days in ICU patients and 4 days in ward patients PE: 8% central, 77% segmental, 15% subsegmental High d-dimer levels, low lymphocytic count associated with thromboembolic manifestations
Wichmann et al. Germany [38]	Autopsy study COVID-19 according to +ve RT-PCR	12 pts	33.3%	DVT in 7 of 12 patients (58%) in whom venous thromboembolism was not suspected before death In all patients, SARS–CoV-2 RNA was detected in the lung at high concentrations 5 of 12 patients demonstrated high viral RNA titers in the liver, kidney, or heart
Klok et al. Netherlands* [39]	Retrospective cohort - ICUs in 3 hospitals	184 pts	35.3%	Increasing follow-up from 7 to 14 days increased the incidence of PE from 13.6 to 35.3% PE: 70.8 segmental or more proximal arteries, 29.8% subsegmental arteries
Bompard et al. France [40]	Retrospective cohort 2 Hospitals	135 pts COVID-19 + CTA	23.7%	Sixty-three pts (47%) were outpatients seen at the emergency department Fifteen PE were diagnosed in outpatients at initial presentation whereas the remaining 17 were diagnosed in patients who had presented clinical deterioration during hospitalization PE: 31% proximal, 56% segmental, 13% multiple sub segmental pulmonary arteries 4 patients with PE died (13%) within a median of 26 days All patients received prophylactic anticoagulation
Thomas et al. UK [41]	Retrospective Single center	63 pts	7.9%	PE, 20% sub-segmental, 40% segmental, 20% multiple segmental and 20% in a main pulmonary artery None of the patients that developed thrombosis had a history of either active cancer or VTE Very short follow-up (median 8 days)
Poyiadi et al. USA [42]	Retrospective Multicenter	328 pts COVID-19 + CTA	22%	PE: 51% segmental, 31% lobar, 13% central, 5.5% subsegmental 28/122 (23%) of all patients that were on venous thromboprophylaxis developed a PE Statin therapy associated with lower and BMI > 30 kg/m^2^, d-dimer of 6 μg/mL with higher risk of developing PE
Galeano-Valle et al. Spain [43]	Prospective Single center	785 pts COVID-19	1.9%	PE: 40% had intermediate–high risk PE and 60% patients had low risk PE Non-ICU setting, low severity of illness
Stoneham et al. UK [44]	Retrospective 2 hospitals	274 pts Confirmed or highly suspected COVID-19	5.8%	White cell count, d-dimer, and fibrinogen associated with the occurrence of VTE in COVID-19 patients Almost all patients had an abnormal d-dimer result at baseline, defined as a d-dimer > 0.5 µg/mL Three patients were described to have resistance to anticoagulation
Lax et al. Austria [45]	Autopsy study	11 pts	100%	Ten of the 11 patients received prophylactic anticoagulant therapy; Venous thromboembolism was not clinically suspected antemortem in any of the patients Thrombosis of small and mid-sized pulmonary arteries was found in various degrees in all 11 patients and was associated with infarction in 8 patients

ARDS: acute respiratory distress syndrome, CTA: angiographic computed tomography, DVT: deep venous thrombosis, ICU: intensive care unit, PA: pulmonary artery, PE: pulmonary embolism, pts: patients, MV: mechanical ventilation, RT-PCT: real-time reverse transcriptase polymerase chain reaction, UK: United Kingdom

* Same cohort, analysis updated to increase the follow-up period from 7 to 14 days

The incidence of PE in hospitalized patients with COVID-19 has been reported to be around 1.9–8.9% [29, 33, 43, 44]. Again, the retrospective nature of the reported cohorts and the relatively short periods of follow-up may have underestimated the real incidence of PE. Critically ill COVID-19 patients requiring ICU admission seem to be at a higher risk of thromboembolic complications, especially PE, which may occur in up to 26.6% of these patients [36]. In a prospective observational study of 150 patients admitted to four ICUs in two French hospitals, PE occurred in 16.7% of patients despite thromboprophylaxis [31]. The authors also reported that thromboembolic events were more common in COVID-19 patients with acute respiratory distress syndrome (ARDS) compared to a propensity score-matched historic ARDS cohort, underscoring the unique procoagulant effect of COVID-19 compared to other ARDS etiologies. A retrospective cohort of 184 patients with COVID-19 admitted to ICUs in three hospitals in the Netherlands reported that PE occurred in 13.6% of the patients despite anticoagulant therapy [32]. Interestingly, the incidence of PE increased to 33.3% when the follow-up was increased from 1 to 2 weeks [39], at a time when heightened awareness of the common occurrence of PE may have led to a higher index of suspicion and more diagnostic procedures to detect this complication. Likewise, Poissy et al. showed that 20.6% of patients admitted to a French ICU had PE within a median of 6 days following ICU admission despite anticoagulation [35]. These authors also found that the frequency of PE in COVID-19 patients was twice as high as the frequency in patients admitted to the ICU in a control period as well as in 40 ICU patients with influenza.

The paucity of deep venous thrombosis (DVT) or other sources of VTE in COVID-19 patients with PE [31] may suggest that, at least in some cases, pulmonary thrombosis rather than embolism is the underlying lesion in these patients. Nonetheless, autopsy of 12 consecutive patients admitted to an academic medical center in Germany revealed DVT in 7 of 12 patients (58%) in whom VTE was not suspected before death [38]. The prevalence of DVT in COVID-19 patients may, therefore, have been underestimated because of the lack of repeated screening in these patients. The authors also reported that PE was the direct cause of death in one-third of patients, confirming the clinical relevance of this complication [38]. Another autopsy study in 11 COVID-19 patients found that death may be caused by the thrombosis observed in segmental and subsegmental pulmonary arterial vessels in all patients, despite the use of prophylactic anticoagulation [45]. Taken together, whereas the current evidence may not support the routine screening for DVT in COVID-19 patients as recommended by the International Society of Thrombosis and Haemostasis (ISTH) [49], high degree of clinical suspicion in diagnosing DVT should be adopted in these patients based on both clinical manifestations and laboratory data. Patients with otherwise unexplained deterioration in the clinical picture, those with local signs and symptoms of DVT, together with markedly elevated d-Dimer levels may be good candidates for diagnostic ultrasound assessment.

## Pathophysiology of PE in COVID-19

### The hypercoagulable state in COVID-19

The hypercoagulable state in COVID-19 was confirmed in a study by Han et al., in which higher levels of d-dimer, fibrinogen, and fibrinogen degradation products [46], prolonged prothrombin time (PT), international normalized ratio (INR), and thrombin time (TT) were also noted in patients with COVID-19 [47]; these abnormalities have been associated with poor prognosis in patients infected with SARS-CoV-2 [48]. Oudkerk et al. suggest that the very high d-dimer levels observed in COVID-19 patients are not only secondary to systemic inflammation, but also reflect true thrombotic disease, possibly induced by cellular activation that is triggered by the virus [50]. Furthermore, Cui et al. demonstrated that a cut-off value of 3.0 μg/mL for d-dimer had sensitivity, specificity and negative predictive values of 76.9%, 94.9% and 92.5% to predict VTE, respectively [51]. After receiving anticoagulant therapy, the level of d-dimer decreased gradually, showing that d-dimer levels may not only predict thrombosis but also monitor the effectiveness of anticoagulant therapy. Nonetheless, d-dimer levels may not be a reliable predictor of VTE but rather a marker of poor overall outcome [4, 9] [52]. Indeed, Lionard-Lorant et al. showed that d-dimer greater than 2660 μg/L is highly sensitive (100%, 95% CI 88–100) but not specific (67%, 95% CI 52–79) to detect PE in COVID-19 patients [30]. Therefore, routine screening for VTE based on elevated d-dimer levels was not recommended in the most recent guidelines of the ISTH [49].

Viral infections may predispose to VTE [51], activating systemic inflammatory response that in turn causes an imbalance between procoagulant and anticoagulant effects [52]. Coagulation pathways and immune system are strictly linked. Clot formation should limit the loss of blood and immune components [53]. Meanwhile, a blood clot can slow down microorganism invasion of the circulation [53]. Indeed, immunocompromised individuals have been suggested to have had less pulmonary complications when infected with COVID-19 [53]. Thrombin and platelets play a key role in the relationship between immune system and coagulation. On the one hand, thrombin directly links the clotting pathways to the innate immune response [54]. On the other hand, various granular constituents of the platelets show antimicrobial and chemotactic properties [55]. The interaction between coagulation and inflammatory pathways in the bronchoalveolar compartment, also known as the “bronchoalveolar hemostasis” [56] could partially explain thrombotic complications in COVID-19 patients.

### Phenotypes and possible pathophysiologic mechanisms

At least two main phenotypes of COVID-19 patients with thrombotic lung injury can be identified: patients affected by “ordinary” VTE and patients showing pulmonary microthrombosis (PMT). Since DVT or other sources of VTE were not consistently found in COVID-19 patients with PE, PMT could be the result of local hypercoagulability rather than secondary to embolization from the lower limbs [53]. Formation of thrombi in the microvasculature may be a part of the physiological effort to limit the viral load. Indeed, viral invasion induces an intense inflammation of the lungs which in turn triggers a local activation of hemostasis driven by the interaction between platelets and endothelium [53]. It has been speculated that a possible cornerstone of microthrombi generation during COVID-19 is related to endothelial cells’ dysfunction [57, 58].

Interestingly, the coagulation activation pattern in COVID-19 ARDS patients in the ICU was not the same as in non-COVID-19 ARDS patients [31]. Whereas d-dimers levels were less elevated, PT, activated partial thromboplastin time (aPTT), and AT were within normal ranges, and fibrinogen was higher. This pattern differs also from that observed in patients with septic shock, who frequently develop disseminated intravascular coagulation (DIC) [59], Helms et al. reported that no COVID-19 patient was diagnosed with DIC using ISTH “overt” score [31]. The underlying mechanisms of COVID-19-induced coagulopathy may be, therefore, different from that reported in other patients with sepsis. This may also explain the different phenotypes observed in COVID-19 patients, with predominant thromboembolic manifestations rather than bleeding tendency.

**Fig. 1:**
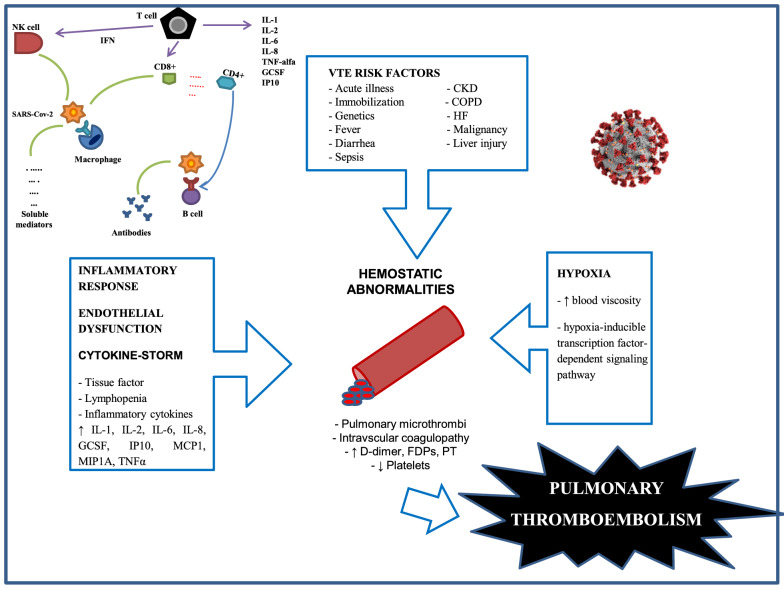
Schematic representation of the possible pathophysiologic mechanisms underlying pulmonary embolism (PE) in patients with coronavirus disease-2019 (COVID-19). CD: CD receptor, CKD: chronic renal failure, COPD: chronic obstructive pulmonary disease, FDP: fibrin degradation products, GCSF: granulocyte-colony stimulating factor, HF: heart failure IFN: interferon, IL: interleukin, IP: interferon-gamma induced protein, MCP: monocyte chemotactic protein, MIP: macrophage inflammatory protein, NK: natural killer cells, PT: prothrombin time, SARS CoV-2: acute respiratory syndrome coronavirus 2, TNF alpha: tumor necrosis factor alpha

Several mechanisms may contribute to a hypercoagulable state [60] and PMT during COVID-19 [46] (Fig. 1). First, the direct and indirect pathologic consequences of COVID-19, such as severe hypoxia, preexisting comorbidities, and associated organ dysfunction can predispose to hemostatic abnormalities, including DIC [47]. Hypoxia can predispose to thrombosis by increasing blood viscosity and via a hypoxia-inducible transcription factor-dependent signaling pathway [61]. The risk of VTE is also associated with individual patient-related risk factors, such as age, immobilization, obesity, past history of personal or familial VTE, cancer, sepsis, respiratory or heart failure, pregnancy, stroke, trauma, or recent surgery. ICU-specific risk factors may also contribute to this risk, including but not limited to sedation, immobilization, vasopressor administration, and use of central venous catheters [60]. Second, endothelial dysfunction, von Willebrand factor (vWF) elevation, Toll-like receptor activation, and tissue-factor pathway activation [62] may induce proinflammatory and procoagulant effects through complement activation and cytokine release [50], resulting in a dysregulation of the coagulation cascade with the subsequent formation of intra-alveolar or systemic fibrin clots. This may be explained, at least in part, by the increased plasminogen activator inhibitor 1 (PAI-1) levels with subsequent decrease of the fibrinolytic activity in these patients [63]. Helms et al. analyzed the occurrence of thromboembolic events in all patients admitted to four French ICUs for COVID-19-associated ARDS [31]. They noted that vWF activity and vWF antigen (vWF:Ag) were considerably increased, as was factor VIII. Furthermore, 50 of the 57 patients tested (87.7%) had positive lupus circulating anticoagulants during their ICU stay. Third, the release of high plasma levels of proinflammatory cytokines (IL-2, IL-6, IL-7, IL-8, granulocyte colony-stimulating factor, interferon gamma-induced protein 10 (IP10), monocyte chemotactic protein-1 (MCP1), macrophage inflammatory protein 1A (MIP1A) and tumor necrosis factor (TNF-α)—the so-called “cytokine storm”, which is a common feature of sepsis—cause secondary development of hemophagocytic lymphohistiocytosis with activation of blood coagulation, increased risk of intravascular microthrombosis and secondary local consumption coagulopathy [50], promoting the occurrence of VTE. Finally, the interactions between different types of blood cell (macrophages, monocytes, endothelial cells, platelets and lymphocytes) could play a critical role in the procoagulant effect of viral infections. For example, platelet activation upon antigen recognition may facilitate the pathogen’s clearance by white blood cell activation and clot formation [62]. This may be modulated by the neutrophil extracellular traps (NETs), which are induced by platelets and play an important role in sepsis-associated hypercoagulability [64]. In agreement with this assumption, Middeldorp et al. found that white blood cell count, higher neutrophil-to-lymphocyte ratio and a higher d-dimer level are independent risk factors associated with VTE [37].

### The role of endothelial injury

Endothelial cells represent almost a third of the cells in the broncho-alveolar units [65]. Endothelial dysfunction refers to a systemic condition in which the endothelium loses some its physiological properties such as promoting vasodilation, fibrinolysis, and anti-aggregation [65]. This condition could be induced in COVID-19 patients through general and virus-related factors. Comorbid conditions, such as hypertension, diabetes and acute kidney injury are usually linked to endothelial damage and may, therefore, promote COVID-19-related complications [65]. Virus-related factors may also induce endothelial damage through direct viral penetration in endothelial cells, the effects of cytokines on the endothelium, and the release of von Willebrand factor by endothelial cells. Endothelial cells possess the key receptors for the SARS-CoV-2 (i.e., the angiotensin-converting enzyme-2 receptors), that facilitate viral penetration [66]. They also express other receptors shared with SARS-CoV-2, such as serine protease 2 and sialic acid receptors [58]. Accordingly, endothelial cell infection results in some cytopathic modifications following viral penetration. In particular, vascular obliteration and thrombosis of small and middle size vessels are common findings in PMT secondary to COVID-19. Furthermore, the proinflammatory cytokines released in patients with COVID-19 promote vascular endothelial cell apoptosis, PMT, vascular leakage, alveolar edema, and ultimately hypoxia [66]. Proinflammatory cytokines can also increase the expression of adhesion molecules that in turn results in endothelial activation, procoagulant effects and pro-adhesive changes [67]. All these molecular changes can impaire microvascular flow and, consequently, alter ventilation/perfusion ratio. It has been also postulated that endothelial damage and PMT could be induced by an imbalance between insufficient ADAMTS-13 (a disintegrin and metalloproteinase with a thrombospondin type 1 motif, member 13) and excessive exocytosis of ultra large von Willebrand factor multimers (ULVWF) from Weibel–Palade bodies present in endothelial cells [57]. ULVWF are anchored to the endothelial surface and can recruit platelets inducing microthrombogenesis [57]. Subsequently, platelets are rapidly activated causing platelet aggregation and leukocytes recruitment in a P-selectin-dependent manner [57]. These aggregates continue to grow until they become sufficiently large to induce extended PMT.

## Therapeutic implications

**Fig. 2:**
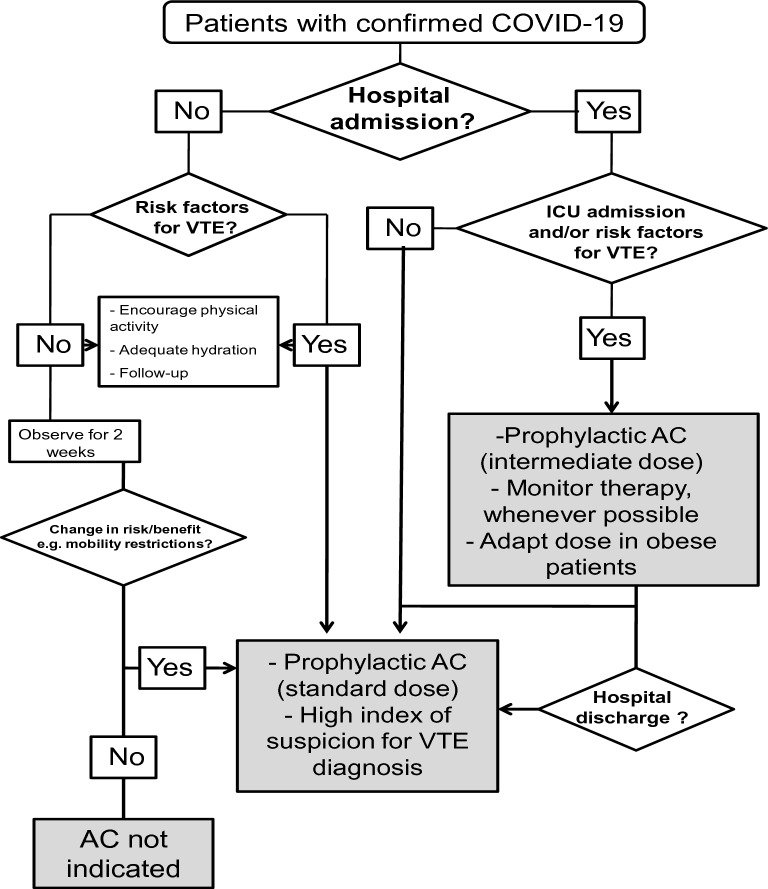
Flow diagram of the recommended procedure for initiating thromboprophylaxis in patients with coronavirus disease-2019 (COVID-19). The choice of the appropriate method for anticoagulation (AC) should be based on individual risk/benefit assessment (see text for details). COVID-10: coronavirus disease-2019, VTE: venous thromboembolism

The accumulating evidence suggests that PE is a significant complication in patients with COVID-19, so that indications and modalities for prophylactic and therapeutic use of antithrombotic agents should be revisited. Preliminary data from 449 consecutive patients with severe COVID-19 demonstrated that prophylactic doses of heparin were associated with improved survival in a subgroup of patients meeting criteria for sepsis-induced coagulopathy or with markedly elevated d-dimer levels [68]. The ISTH and the American Society of Hematology (ASH) [47, 49, 60, 69] have recently recommended that a prophylactic dose of LMWH (40 mg qd) [70] or subcutaneous unfractionated heparin (5000 IU tid)—should be started in all suspected or confirmed COVID-19 patients admitted to the hospital. In patients with known heparin-induced thrombocytopenia, fondaparinux [70, 71], which was found to be effective in reducing sepsis-derived coagulopathy in an animal model [72], should be used. If pharmacological prophylaxis is contraindicated, mechanical VTE prophylaxis (e.g., intermittent pneumatic compression) should be considered in immobilized patients [47]; combined pharmacologic and mechanical prophylaxis is not generally recommended [71]. Although limited data are available, it is reasonable to consider pharmacological thromboprophylaxis in patients admitted to hospital with COVID-19 infection, even in pregnant women, since they are likely to be at an increased risk of VTE [47]. The use of and intermediate dose of LMWH (e.g., enoxaparin 4000 IU subcutaneously every 12 h) can be considered on an individual basis in patients with multiple risk factors for VTE [73] and in critically ill patients due to the higher incidence of PE in this population [29–41]. In obese patients, higher weight-based doses may be needed, with doses of 7500 IU UFH three times daily or enoxaparin 40 mg twice daily [74, 75]. Figure 2 represents a flow diagram of the recommended procedure for initiating thromboprophylaxis in patients with coronavirus disease-2019.

Therapeutic anticoagulation is the cornerstone in the management of patients with PE. Selection of an appropriate agent and correct dosage requires consideration of the underlying comorbidities, such as renal or hepatic dysfunction, thrombocytopenia, and gastrointestinal tract function [47]. Zhai et al. recommend LMWH (e.g., subcutaneous enoxaparin 100 IU/kg, twice daily or 150 IU/kg once daily, or nadroparin 86 IU/kg twice daily) as a first-line treatment [76]. In severe renal impairment, or if it is expected that invasive procedures will be required, intravenous UFH followed by the subcutaneous route is more appropriate, with regular monitoring for anticoagulation dose adjustment. Direct oral anticoagulants (DOACs) are an option only after the acute phase in stable patients, with the well-known benefits of lack of need for monitoring, which facilitates timely discharge from the hospital and outpatient management. However, a potential risk of DOACs, especially in the setting of organ dysfunction, may include clinical deterioration and lack of effective reversal agents at some centers [47]. Geert-Jan Geersing et al. provided a guidance document for switching from vitamin K antagonists (VKAs) to a DOAC in current emergency settings [77]. They underscore the need to switch with care and caution, and the importance of not making this choice just for simplicity, because it may contribute to errors like overlapping periods of anticoagulation, terminating VKA without correctly starting DOACs, and lack of explanation to the patient for the reasons for such drug changes, thus create a potential risk for thromboembolism or bleeding [78]. The use of catheter-directed therapies during the current outbreak should be limited to the most critical situations [47]. Recurrent PE despite optimal anticoagulation and clinically significant VTE in the setting of absolute contraindications to anticoagulation would be among the few scenarios in which placement of an inferior vena cava filter may be considered [47, 79], and even in these cases, anticoagulation should be resumed as soon as feasible. In patients requiring therapeutic doses of LMWH or receiving a DOAC, renal function should be monitored and anti-factor Xa or plasma DOAC levels should be monitored.

Intermediate-risk hemodynamically stable patients (intermediate–low risk or intermediate–high risk PE according to the European Society of Cardiology (ESC) classification; sub-massive PE according to prior classifications) should be managed initially with anticoagulation and close monitoring. If the condition suddenly worsens and there are signs of overt hemodynamic instability (massive or high-risk PE with hypotension or sudden cardiac arrest) and evidence on bedside echocardiography of new onset increased right-ventricular load or pulmonary arterial hypertension, thrombolytic therapy should be initiated urgently [47, 76]. In case of refractory shock or cardiac arrest, extra corporeal membrane oxygenator (ECMO) could be an option, in combination with surgical embolectomy or catheter-directed treatment, as rescue initiatives [76].

Since the procoagulant effect of COVID-19 may extend some weeks after hospital discharge of apparently stable, asymptomatic patients. It would be prudent, therefore, to have a high degree of clinical suspicion of PE in COVID-19 patients readmitted to the hospital after surviving an initial hospitalization. Decisions about extending prophylaxis with LMWH after hospital discharge from acute medical illness should be made by balancing the reduced risk of VTE with the risk of increased bleeding events, including major bleeding. In the absence of high-quality data, pharmacological prophylaxis in this context should be reserved for patients at highest risk, including those with limited mobility and history of prior VTE or active malignancy [47]. As recommended by the Italian Society on Thrombosis and Haemostasis (SISET), prophylactic anticoagulation should be maintained at home for 7–14 days after hospital discharge or in the pre-hospital phase during home self isolation, in case of pre-existing or persisting VTE risk factors (i.e., reduced mobility, body mass index (BMI) > 30, previous VTE, active cancer, etc.) [73].

## Summary and conclusions

Patients with COVID-19 are at increased risk of developing PE which may occur in up to one-third of critically ill COVID-19 patients requiring ICU admission. Thromboprophylaxis should therefore be started in COVID-19 patients admitted to the hospital and intermediate therapeutic doses of anticoagulants can be considered in patients requiring ICU admission or those with multiple risk factors for VTE. Extending thromboprophylaxis after hospital discharge or in the prehospital phase during home self isolation should be done according to a meticulous risk/benefit assessment, balancing the reduced risk of VTE with the risk of increased bleeding events. Therapeutic anticoagulation is the cornerstone in the management of patients with PE. Selection of an appropriate agent and correct dosage requires consideration of underlying comorbidities and organ dysfunction.

## Data Availability

Not applicable.

## Abbreviations

ADAMTS-13: A disintegrin and metalloproteinase with a thrombospondin type 1 motif, member 13
aPTT: Activated partial thromboplastin time
ARDS: Acute respiratory distress syndrome
ASH: American Society of Hematology
BMI: Body mass index
ECMO: Extra corporeal membrane oxygenator
CTA: Computer tomographic angiography
COVID-19: Corona virus disease-2019
DIC: Disseminating intravascular coagulation
DOAC: Direct oral anticoagulants
DVT: Deep vein thrombosis
ESC: European Society of Cardiology
ICU: Intensive care unit
IL: Interleukin
INR: International normalized ratio
IP: Interferon-gamma induced protein
ISTH: International Society of Thrombosis and Haemostasis
I.U.: International unit
IQ: interquartile range
LMWH: Low molecular weight heparin
MCP: Monocyte chemotactic protein
MIP: Macrophage inflammatory protein
NETs: Neutrophil extracellular traps
PMT: Pulmonary microthrombosis
PT: Prothrombin time
PAI-1: Plasminogen activator inhibitor 1
PE: Pulmonary embolism
RT-PCR: Real-time reverse transcription polymerase chain reaction
r-tPA: Recombinant tissue-type plasminogen activator
SARS-CoV-2: Severe Acute Respiratory Syndrome Coronavirus 2
SISET: Italian Society on Thrombosis and Haemostasis
TT: Thrombin time
ULVWF: von Willebrand factor multimers
VTE: Venous thromboembolism
VKA: Vitamin K antagonist
vWF: Von Willebrand factor
vWF:Ag: vWF antigen
